# Development and validation of a photographic food atlas of Middle Eastern Mediterranean diet: Toward improved understanding of traditional healthy and sustainable diets

**DOI:** 10.3389/fnut.2022.982420

**Published:** 2023-01-11

**Authors:** Manal Badrasawi, Mohammad Altamimi, Souzan Zidan, Anne-Kathrin Illner, Krasimira Aleksandrova

**Affiliations:** ^1^Department of Nutrition and Food Technology, Faculty of Agriculture and Veterinary Medicine, An-Najah National University, Nablus, Palestine; ^2^Department of Nutrition and Food Technology, Faculty of Agriculture, Hebron University, Hebron, Palestine; ^3^Institut Polytechnique UniLaSalle, Université d’Artois, ULR 7519, Equipe PANASH, Beauvais, France; ^4^Department of Epidemiological Methods and Etiological Research, Leibniz Institute for Prevention Research and Epidemiology (BIPS), Bremen, Germany; ^5^Faculty of Human and Health Sciences, University of Bremen, Bremen, Germany

**Keywords:** food atlas, Middle Eastern Mediterranean diet, dietary assessment, portion size, sustainability

## Abstract

**Background:**

Middle Eastern Mediterranean diet (MEMD) is a traditional plant-based diet that is commonly consumed and increasingly popular, but not well studied in nutrition research. To facilitate the dietary assessment of MEMD, we developed and validated a photographic food atlas depicting a variety of foods and dishes consumed in the MEM region.

**Methods:**

The photographic food atlas included 1,002 photos of 400 types of foods and traditional dishes photographed characterizing MEMD. Foods and dishes were prepared by a professional cook and were subsequently photographed as a series of photos depicting portion size options. In a validation study, 45 individuals aged 20–50 years were recruited to assess portion size estimation of 25 representative food-photo series for each item. The validity of portion size estimation was assessed by comparing actual and reported estimates using Pearson or Spearman correlation tests. Sizes of the differences between estimated portions and the actual served portion sizes were calculate as mean differences and standard deviations.

**Results:**

In the validation study, there was a strong correlation (*r* > 0.7) between estimated portion size of actual foods for 7 food items, such as pita bread, milk, *labneh*, and tomatoes, a moderate correlation (< 0.5 | *r* | < 0.7) for 12 items, such as meat, chicken, and grapes, and weak correlation (*r* < 0.3) for 6 items, such as seeds. Underestimation of portion sizes was more commonly observed for food items quantified when using “grams” or “milliliters” as a unit of measurement. In contrast, when household measurements were used, the participants tended to overestimate the portion sizes of respective foods and dishes.

**Conclusion:**

We developed and validated a photographic food atlas depicting a wide variety of foods and dishes typical for the MEMD. The application of the photographic food atlas may facilitate the accurate assessment of adherence to MEMD and support the understanding of its health and sustainability aspects. Further methodological work is warranted to extend the list of food items and to evaluate the validity of the food atlas among larger and more heterogeneous groups of participants.

## Introduction

Over the last decade, there has been an increased recognition on the role of sustainable food policies and the resulting impacts on the environment and health outcomes (1, 2). Sustainable healthy diets have been defined as dietary patterns that promote all dimensions of individuals’ health and wellbeing; have low environmental pressure and impact; are accessible, affordable, safe; and culturally acceptable (1). Traditional plant-based diets such as the Middle Eastern Mediterranean diet (MEMD) may cover both health and sustainability aspects; however, this type of diet has been largely understudied in nutrition research. There is a need of affordable, practical, and culturally appropriate methods for dietary intake assessment tailored to the cultural context of the EM region. In this context, retrospective dietary assessment methods such as food frequency questionnaires (FFQs) or 24-h dietary recalls are deemed as more feasible and culturally appropriate for collecting data in large number of individuals over prospective methods such as weighed food records. However, dietary recall methods rely on respondents’ memory on the type and portion size of consumed foods (3). Therefore, they are prone to reporting bias, such as unintentional under-reporting of sporadically consumed food items (e.g., snacks) or intentional under-reporting of unhealthy foods while over-reporting healthy foods (4, 5). Estimation of portion size of complex foods, such as meals and dishes has been especially challenging (4, 6–8). To assist study participants in portion size estimation, a variety of tools have been developed including household measures, food models, and food photographs (9–11). Since eating habits vary across populations, portion size estimation tools are expected to take into account the local context (12). So far, a variety of photographic food atlases have been developed in populations across the globe including Japan (7), Malaysia (9), South Korea (13), Greece (10), China (6), Sri Lanka (14), Austria (15), and UK (4). MEM countries (including Palestine, Levant region; Syria, Lebanon, Jordan, and Iraq) share a similar food culture and it has been increasingly spread across the globe following dynamic migration rates and overall globalization in the last decades. This has led to an increased popularity and consumption of various foods and dishes typical to the MEMD diet in countries around the world, particularly in North America and Europe. However, assessment of adherence to MEMD in regional and international research context has been limited by the lack of photographic food atlas to assist portion size estimation. We therefore aimed to develop and validate a photographic food atlas for portion size assessment of a variety of foods and dishes typical for the MEMD.

## Materials and methods

**FIGURE 1:**
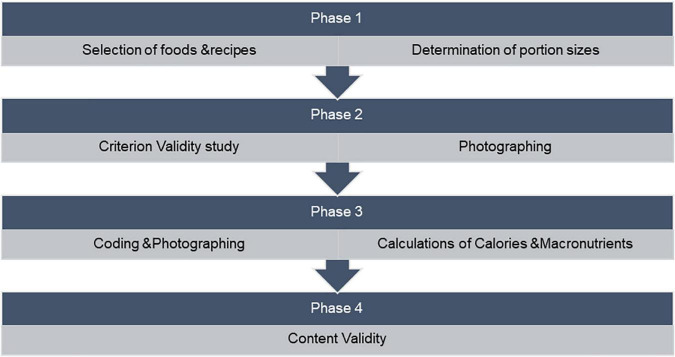
Phases of the development and validation of photographic food atlas.

The format of the photographic food atlas was chosen based on previously developed tools (3, 12, 16–18). The development and validation of the photographic food atlas underwent a multistep approach that included: (1) selection of foods and recipes and portion size measurement; (2) criterion validity assessment; (3) coding of photos, photographing, and macronutrient composition; and (4) content validity assessment (Figure 1).

### Phase 1: Selection of foods and recipes, and portion size measurement

Foods that are typically consumed in the MEM region were identified after reviewing existing food composition tables (16, 17) and exchange lists for traditional dishes (17). Restaurant menus and local cookbooks were additionally reviewed to select commonly served local foods and dishes. In a convenient sample of 100 participants aged 20–50 years, a 24-h dietary recall was administered to identify additional custom local meals and dishes. Based on the reported 24-h dietary recalls, several traditional dishes were identified including *lack seeds dessert*, a local *rice-based vegan dish cooked with green fava beans*, and *a local herbal drink*. A focus group of 15 local experts in nutrition research [8 registered dietitians, 3 university professors, 2 professional chefs, 2 co-investigators (MB and MA)] was formed to discuss and approve the final list of food items to be included in the food atlas. As a result, 400 photos of commonly consumed traditional MEMD food items were produced. These photos were categorized into 25 representative food items based on the following criteria; (1) foods that share same food group; (2) foods that share same nutritional properties; (3) foods that share the same way of presentation (i.e., freekeh “a traditional cereal made from green durum wheat,” pita bread, and taboon bread “Levantine flatbread baked in a *taboon* or *tannur* clay oven”). The portion sizes were expressed as weight (i.e., grams), volume (i.e., milliliters), or in household measurements (i.e., cups, tablespoons, scoops, etc.).

### Phase 2: Criterion validity assessment

The list of 25 representative foods, covering the basic food groups and traditional Levantine and Palestinian dishes were included in criterion validity study conducted at the Department of Nutrition and Food Technology at An-Najah National University, Palestine. The following main food groups were assessed: bread, vegetables, fruits, milk and dairy products, meats, fat and oil, cereals, legumes, nuts, and miscellaneous. The traditional Levantine and Palestinian dishes included: main dishes, appetizers and soups, salads, pastries, desserts, and juices. To validate the photos, 45 lay participants aged >18 years with no formal education in nutrition and food processing were recruited. The age of the participants ranged from 18 to 54 years (mean ± SD: 34 ± 8) and 75% were females. In terms of employment status, 20% were students, 53% were administrative employees, while 27% were technicians. Majority of participants (65%) reported to have an urban residency. Participants were informed about the study design and objectives and a written informed consent was obtained. The study protocol was approved by the Institutional Review Board at An-Najah National University (Ref: Feb. 2019/4). The food dishes were cooked according to a standard recipe by a professional cook. Prepared food was measured on an electronic kitchen weighing scale SF-400 with a precision of 1 g and a maximum capacity of 10 kg, calibrated for the serving plate weight. Food items were served on a white standard shallow plate (diameter = 24 cm) or small white shallow plate (diameter = 18 cm). For the assessment of beverages several types of glassware are presented. Two different types of glassware were used (100 and 200 ml). All food photos were taken according to an official photography protocol, which was approved by three professional photographers, using a digital camera Canon 6D (Canon U.S.A. Inc., Japan). All photos were taken using a lead photo box and a standard lighting setting at analogous conditions: 45° angle, and a distance of 50 cm above the plate. The photos were always taken with a fixative camera on a tripod above the plate at eye level of a sitting person of average height and were color-printed on A4 hard copy. Two sets have been prepared including actual foods and dishes and food photos arranged with codes. The researchers gave each participant two sheets containing a list of 25 food items, where one sheet was intended for estimating the real food and the other sheet for estimating portion sizes based on the food photos. After completing a questionnaire to assess socio-demographic characteristics, participants were asked to estimate the size, weight, and volume of actual foods and foods displayed in the photos and to record their estimation on the data sheet that they were given at the beginning of the session. After the data collection, a validation analysis was performed to determine the agreement between the estimated values of actual foods and the food photos (19).

### Phase 3: Coding of photos, photographing, and macronutrient composition

All food items were numerically given 4-digit codes. Food and traditional dishes were presented in three portion sizes (small, medium, and large) according to the Palestinian and Levantine style. The dishes in different portion sizes were measured twice, in weight or volume and in household measurements. Thereafter, photos were taken for each food and traditional dish according to the standard protocol previously described in phase 2. At the end of this phase, calories and macronutrient content were calculated using the available food composition database, which included the food composition tables for Bahrain (16), Lebanon (17), and the food exchange list for Jordan (18). If the data were not available, the nutrient composition was calculated using the NutriSurvey software^®^. The nutrient composition for the basic food items such as fruits, vegetables, popular types of bread, and common cereals were compared cross the above mentioned references to assure the validity of the values. The food atlas was finalized with the assistance of a professional graphic designer.

### Phase 4: Content validity assessment

The draft of the photographic food atlas was circulated among eight experts in the field of nutrition from West Bank, Gaza, Yemen, and Algeria for evaluation of its content validity. Following expert review and discussion, the presentation of nutrient composition data and the number of portions per each food item were revised. No changes were done with regards to the assessment of nutrient content accuracy.

## Statistical analysis

In the validation study, the mean differences and standard deviations were calculated between the recorded and estimated measurements (in weight, volume, or household measurement units). Correlation between the actual food and the food photos were estimated using Pearson correlation for normally distributed variables or Spearman tests for non-parametric data. The strength of correlations was interpreted as follows: <0.9 | *r* | <1.0 = very high, <0.7 | *r* | <0.9 = high, <0.5 | *r* | <0.7 = moderate, and <0.3 | *r* | <0.5 = low or weak (20). The estimates were considered overestimated if the reported portion size was at least 20% higher as the actual portion size, vice versa they were considered underestimated if the reported portion size was at least 20% lower as the actual portion size. All analyses were performed using IBM SPSS Statistics for Windows, Version 23.0 (IBM, Armonk, NY, USA), and *P*-values < 0.05 were considered statistically significant.

## Results

### An overview of the food atlas

A total of 1,002 photos of 400 types of foods and traditional MEM dishes distributed within 16 subsections were included in the photographic food atlas (Table 1). The food atlas was divided into two main sections: (1) “food groups” including 10 basic food groups: breads, vegetables, fruits, milk and dairy products, meats, fat and oil, cereals, legumes, nuts, and miscellaneous; and (2) “traditional Palestinian and Levantine dishes” including 6 subsections: main dishes (i.e., *maqlobah*, *musakhan*, and *koftah*), appetizers and soups (i.e., *qubah*, *samposak*, and *falafel*), salads (i.e., *fatoush* and *taboulah*), pastries (i.e., *nabulsi* cheese, *zatar*, and thyme), desserts (i.e., *konafah* and *baqlawah*), and juices and drinks (i.e., *kharoub* and *tamarinds*).

**TABLE 1:** Type and number of foods and traditional dishes included in photographic food atlas.

Main sections	Subsections	Food varieties	Total number of pictures
Food groups	Bread	10	28
	Vegetables	27	119
	Fruits	26	69
	Milk and dairy products	7	18
	Meats	7	32
	Fat and oil	8	22
	Cereals	9	28
	Legumes	10	30
	Nuts	11	36
	Miscellaneous	9	18
Traditional Levantine and Palestinian dishes	Main dishes	69	375
	Appetizers and soups	31	78
	Salads	20	20
	Pastries	8	33
	Desserts	44	88
	Juices	8	8

**FIGURE 2:**
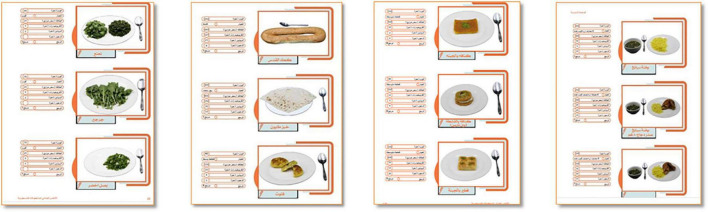
Sample from the developed photographic food atlas, different sections.

The foods and traditional dishes were photographed either as single photos or as a series of photos depicting portion sizes. Photos were combined with brief information and relevant citations on the calories and macronutrient content for each food or traditional dish as shown in Figure 2. Finally, the photos were gathered into a booklet that included a legend with household measurements (see, Table 1).

### Criterion validity study

A total of 25 food items were included in the validation study. Each food item’s name, its portion, and measured weight in grams, milliliters, or household measurements were recorded (see, Table 2). Table 2 shows the correlations between the estimated weights or volumes for the 25 items for both the actual dishes and food photos. Overall, for all food items there have been statistically significant correlations between actual food and reported food intakes. Correlations were particularly strong (*r* > 0.7) for seven food items, namely *freekeh*, pita bread, milk, *labneh*, apples, tomatoes, and *baqlawah*. Moderate correlations (<0.5 | *r* | <0.7) were observed for 12 food items, i.e., *taboon* bread, *labneh* ball, *Nabulsi* cheese, chicken, meat cubes, grapes, olive oil, almonds, *konafah*, *mamoul*, *muttabal*, and lentil soup. The remaining six food items, including seeds, pickles, and salmon fish, showed weak correlations (*r* < 0.3) between actual and reported values. Among various food groups, higher correlation estimates were observed for bread, vegetables (tomato), fruit (apple), meat (fish), Arabic dessert (Konafah), cereals (freekeh), fat (olive). In contrast, low correlations were seen for milk, dairy product (labneh), and nuts.

**TABLE 2:** The correlations between the estimated portion values for real food and food photo using weight and volume.

	Food item	Description/Form	The measurable weight (gram/ml)	Real food estimation Mean + SD/or median (min.–max.)	Food photo estimation Mean + SD/or median (min.–max.)	Correlation coefficient	*P*-value
Bread	Pita bread^1^	Half loaf	55 g	39 (30–150)	32 (20–150)	0.901	<0.01^a^
	Taboon bread^2^	1 ½ of the loaf	60 g	47 (30–150)	45 (40–200)	0.68	<0.01^a^
Vegetables	Tomato	Raw whole tomato	60 g	37 (30–200)	32 (30–200)	0.819	<0.01^a^
	Tomato	Raw, cut into cubes	150 g	98 ± 88	120 ± 79	0.47	<0.05^b^
Fruits	Apple	Raw whole apple	100 g	85 ± 59	84 ± 50	0.75	<0.01^b^
		Raw, cut into cubes
	Grapes	Whole grapes	170 g	97 (50–300)	120 (30–400)	0.523	<0.01^a^
Milk and dairy products	Milk	Fluid	100 ml	117 ± 16.5	112 ± 17	0.771	<0.01^b^
	Labneh^3^	Amorphous	24 g	30 ± 17	28 ± 18	0.772	<0.01^b^
	Labneh ball	Ball	24 g	21 ± 14	18 ± 11	0.602	<0.01^b^
	Nabulsi cheese^4^	Cut into pieces	30 g	30 (20–100)	25 (10–80)	0.582	<0.01^a^
Meats	Chicken	Cooked, cut into quarters	100 g	95 ± 51	98 ± 49	0.694	<0.01^b^
		Cooked, cut into eighths
	Fish	Cooked whole fish	130 g	100 (50–250)	100 (80–300)	0.487	<0.01^a^
	Salmon fish	Cooked, cut into slices	160 g	200 (30–350)	170 (70–500)	0.358	<0.05^a^
	Meat ball	Cooked, shaped into balls	35 g	60 ± 40	58 ± 47	0.482	<0.05^b^
	Meat cubes	Cooked and cut into cubes	45 g	54 ± 55	40.1 ± 56	0.57	<0.01^b^
Fat and oil	Olive oil	Fluid	20 ml	14 (5–50)	20 (10–70)	0.588	<0.01^a^
Cereals	Freekeh^5^	Cooked	100 g	95 ± 16.4	82 ± 42.9	0.77	<0.01^b^
Legumes	Lentils	Cooked	200 ml	100 (30–200)	150 (20–300)	0.586	<0.05^a^
Nuts	Almonds	Raw pieces	40 g	32 (20–70)	35 (10–100)	0.543	<0.01^a^
	Seeds	Raw pieces	20 g	25 (10–75)	25 (10–150)	0.391	<0.01^a^
Miscellaneous	Pickles	Piece	90 g	35 (10–110)	25 (20–120)	0.21	>0.05^a^
Appetizers	Muttabal^6^	Piece	50 g	38 (20–100)	34 (20–150)	0.345	0.05^a^
Desserts	Konafah	Piece	180 g	200 (50–300)	172 (100–400)	0.699	<0.01^a^
	Baqlawah	Piece	20 g	20 (10–70)	20 (10–80)	0.834	<0.01^a^
	Mamoul	Amorphous	40 g	40 (30–100)	35 (20–120)	0.67	<0.01^a^

Descriptive analysis presented in mean ± SD in normally distributed data or median (minimum, maximum) if the data is not normal.

^a^Spearman correlation test; ^b^Pearson correlation test.

^1^A flat breads baked from wheat flour; ^2^traditional bread baked from wheat flour wooden baked; ^3^using yogurt that have been strained to remove most of its whey; ^4^traditional Palestinian goat cheese; ^5^traditional cereals made from green durum wheat; and ^6^an appetizer made from grilled eggplant.

Table 3 shows the correlation coefficients between 12 food photos and actual foods measured using household measurements (i.e., scoop, cup, and tablespoon). Statistically significant correlations (*p* < 0.01) were found for all food items, with a strong correlation (*r* > 0.7) reported for *freekeh*, apples, grapes, and lentil soup. A weak correlation was reported for seeds (*r* > 0.3), while the rest of the items showed moderate correlations (<0.5 | *r* | <0.7). Overall, the reported intakes based on both actual food and food photos were generally overestimated for all food items, except for pita bread and lentil soup. There were no differences in the mean of estimation of food intakes according to socio-demographic status of the participants.

**TABLE 3:** The correlations between the estimated portion values for real food and food photo using household measurements.

Foods and traditional dishes		Description/Form	The measurable weight (gram/ml)	Real food estimation Mean ± SD	Food photo estimation Mean ± SD	Correlation coefficient	*P*-value
Bread	Pita bread^1^	Half loaf	13 cm	12 (3–20)	10 (2–20)	0.68	<0.01^a^
Vegetables	Tomato	Raw, cut into cubes	1 Cup	1 (0.5–3)	1.5 (0.5–3)	0.657	<0.01^a^
	Lettuce	Raw shredded	1 Cup	1.5 (0.5–3)	1 (0.5–3)	0.61	<0.01^a^
Fruits	Apple	Raw whole apple	1 Cup	1 (0.5–4)	1.5 (0.5–4)	0.94	<0.01^a^
		Raw, cut into cubes
	Grapes	Raw whole grapes	1 Cup	1.5 (1–4)	1.5 (1–4)	0.91	<0.01^a^
Milk and dairy products	Labneh^2^	Amorphous	1 Tablespoon	2 (1–3)	1.5 (0.5–4)	0.629	<0.01^a^
Fat and oil	Olive oil	Fluid	1 Tablespoon	1.1 ± 0.3	1.3 ± 0.7	0.688	<0.01^b^
Cereals	Freekeh^3^	Cooked	2 Scoop	2 (1–5)	1 (1–5)	0.87	<0.01^a^
Legumes	Lentils	Cooked	3 Scoop	2 (1–6)	2 (1–8)	0.78	<0.01^a^
Nuts	Almonds	Raw pieces	3 Tablespoon	3.6 ± 1.4	2.8 ± 1.1	0.65	<0.01^b^
	Seeds	Raw pieces	3 Tablespoon	4 (1–9)	3 (1–13)	0.321	<0.01^a^
Appetizers and soups	Muttabal^4^	Amorphous	2 Tablespoon	3 (1–5)	2 (1–6)	0.684	<0.01^a^

^1^A flat breads baked from wheat flour; ^2^yogurt that have been strained to remove most of its whey; ^3^traditional cereals made from green durum wheat; and ^4^an appetizer made from grilled eggplant.

Descriptive analysis presented in mean ± SD in normally distributed data or median (minimum, maximum) if the data is not normal.

^a^Spearman correlation test; ^b^Pearson correlation test.

## Discussion

In this methodological study, we developed and validated a photographic food atlas for portion size assessment of a variety of foods and dishes typical for the MEMD. It can be used as a tool to facilitate the conduct of dietary assessment surveys in Palestine and Levant region, but also can find application in studies that aim to capture adherence to MEMD in various populations across the globe.

Methodological research in the field of dietary assessment has been primarily focused on assisting study participants in describing the consumed food volume, size, and weight, thereby improving accuracy and precision of diet records (21). These can be achieved, to some extent, by using household measurements. Practically, food estimation of this type needs training and is time consuming, especially when larger groups are recruited or surveyed. Using photographic materials to aid participants’ intake and portion size estimation has been suggested as an efficient approach to minimize the error in reported amounts of consumed and estimated food intake (22). Such tools have also proven useful to deal with reporting bias. In this context, previous study done by Ptomey et al. (5) demonstrated the utility of using digital photographs in combination with dietary recalls (DP + *R*) in assessing energy intake in overweight participants that tend of underreport dietary intakes.

So far, a number of photographic food atlases has been developed in various populations across the globe (3, 12, 23–25). Validation of these tools have been assessed by weighing the food items before and after meals (4, 26, 27). Despite useful, this approach is overwhelming for researchers and participants. Comparison of the portion sizes of the food photos with household measurements people are familiar with such as mugs, teacups, glasses, spoons, and other tableware has proven as a more feasible approach (7). Using a common object of reference i.e., knife, a can of beverage, or a pack of gum has been further suggested (6). Using series of photos has been shown to serve as a more accurate method compared to using one photo (9, 14).

The development and validation of the current food atlas was done using a carefully designed and implemented multi-component and multi-stage methodology. The development phase included identifying food products from various sources and conducting a dietary assessment study using 24-h dietary recalls to thoroughly investigate local food options. The validation phase assessed the content and criterion validity of the food atlas. Despite conducted in academic setting, the validation study was based on a sample that was representative to the general Palestinian population in terms of knowledge and perception to the food items (28). Similarly, Wong and Wong (9) have recruited participants with no nutritional experience and not following a special diet. Other studies targeted specific groups of participants such as students, young children, obese adults, or elderly following more strict inclusion criteria (14, 16).

In the current study, underestimation was more common when the measurement units were weight (in grams) or volume for liquids (in ml) as compared to household measures. The underestimation ranged from 1 to 53 g, which represented 1.6% of traditional bread and 33% of fish. These differences in estimation values are higher than those reported by other studies. For instance, Nicklas et al. (29) reported a 10.6-gm difference at 5% of the actual weight. This phenomenon in our data can be partly explained by the fact that the mainly consumed foods are usually sold in larger quantities at the Palestinian market. For example, grapes, tomatoes, and seeds are sold in kilograms rather than grams. On the other hand, underestimation based on food photos occurred in 18 food items (72%) compared to seven items which were overestimated with a range of 2 to 50 g. This contrasts with the findings of UAE researchers who used food photos to estimate portion sizes. They found that the majority of the participants tended to overestimate food portions by 9.5% (for fries)–90.9% (for spicy rice) when photos were used (12). The validity of the methods was tied to how close the estimated values were to the values of the actual food (30). It is noteworthy that the overestimated values—whether based on actual dishes or food photos—were observed for milk, *labneh*, cheese, fish, meat, and seeds. This could be because these items are sold in bulk or served as part of a cooked dish. In contrast, the opposite pattern was observed in the results involving household measurements where most of the food items were overestimated. In fact, only 2 items were underestimated (16.7%), while 10 items were overestimated when real food was used. With food photos, four items were underestimated (33.3%) compared to eight that were overestimated. This was partly due to the participants’ familiarity with household measurements as well to the fact that household measurements (i.e., cups, scoops, and tablespoons) are larger in weight or volume than the scale in grams or milliliters so the range of estimation errors was much less. Overestimation ranged from 0.1 tablespoon to 0.5 cup for real food, and from 0.6 tablespoon to 0.6 cup for food photos. In general, these ranges of estimation error are acceptable and were found for most of the food items.

Similarly, it was found that the average percentage of correct photos chosen by the participants was 60.3% (3). This is another consistent trend that needs to be considered, not only for the validation process, but also for planning and estimating food intake. Researchers and dieticians should take into account that when food portions are recommended to individuals in grams or milliliters, there is a likelihood that the portions may be underestimated. In contrast, when the recommendations are made using household measurements, the portions may likely be overestimated.

This study resulted in the production of photographic food atlas, which is considered the first food atlas to be developed in the Palestine and Levant region containing the most consumed foods and traditional dishes. On the other hand, this study has some limitations that should be acknowledged. First, the validation study has been restricted to adult participants and may not be applicable to children and adolescents. Second, the format of presentation of some food items may not have been optimal. For example, nuts were presented on plates, however, they are not typically served and consumed on a plate. Third, the food atlas may not have included the full variety of foods and dishes consumed as part of the MEMD. Furthermore, it did not include different portion sizes for some foods (e.g., soups and salads). Finally, the validation study was based on a small sample of participants and future studies are warranted based larger and heterogeneous study groups, to allow accounting for potential influence of various factors on the dietary assessment.

## Conclusion

We developed and validated a photographic food atlas depicting a wide variety of foods and dishes typical for the MEMD. The application of the photographic food atlas may facilitate the accurate assessment of adherence to MEMD and support the understanding of its health and sustainability aspects. Further methodological work is warranted to extend the list of food items and to evaluate the validity of the food atlas among larger and more heterogeneous groups of participants.

## Data availability statement

The original contributions presented in this study are included in the article/supplementary material, further inquiries can be directed to the corresponding author.

## Ethics statement

The studies involving human participants were reviewed and approved by the An-Najah National University IRB Committee (Ref. Mas Oct. 2020/4). The patients/participants provided their written informed consent to participate in this study.

## Author contributions

MB had written the study proposal and protocol and supervised the data analysis. MA participated in the study protocol revision and wrote the first draft of the manuscript. SZ had participated in writing the first draft. A-KI and KA consulted the data analysis and interpretation of data and substantively revised the manuscript. All authors read and approved the final version of the manuscript.
